# In vivo Fluorescent Molecular Imaging Using Nanobodies Labeled with Next-Generation FNIR-Tag-Dyes

**DOI:** 10.21203/rs.3.rs-8898776/v1

**Published:** 2026-03-03

**Authors:** Dora M. Chigoho, Marcus C.M. Stroet, Jelena Saliën, Sofie Pollenus, Dong-Hao Li, Martin Schnermann, Sophie Hernot

**Affiliations:** 1 Laboratory for Molecular Imaging and Therapy (MITH), Vrije Universiteit Brussel, Laarbeeklaan 103, 1090 Brussels, Belgium.; 2 Chemical Biology Laboratory, Center for Cancer Research, National Cancer Institute, Frederick, Maryland 21702, United States.

**Keywords:** Single-Domain Antibodies, Fluorescence Molecular Imaging, Near-Infrared Fluorescent Dye, FNIR-Tag, Pharmacokinetics

## Abstract

**Purpose:**

Fluorescently labeled Nanobodies (Nbs) provide rapid, specific, and high-contrast molecular imaging capabilities, making them well suited for applications such as fluorescence-guided surgery. As dye properties can sbstantially influence tracer performance, this study evaluated three next-generation FNIR-Tag dyes, each conjugated to an anti-EGFR Nb.

**Procedures:**

**The anti-EGFR Nb 7D12 was conjugated** to FNIR-Tag-1.0, FNIR-Tag-766 or FNIR-Tag-804, and characterized in vitro. In vivo imaging and biodistribution studies were performed to assess pharmacokinetics, tumor uptake, tumor-to-background ratios, contrast-to-noise ratios, and renal clearance.

**Results:**

All three Nb-based tracers exhibited similar overall pharmacokinetic behavior, characterized by fast tumor accumulation, rapid clearance from blood and non-target tissues, and predominant renal elimination. 7D12–FNIR-Tag-1.0 demonstrated slightly higher tumor uptake, whereas 7D12–FNIR-Tag-766 produced marginally improved tumor-to-background and contrast-to-noise ratios. In contrast, 7D12–FNIR-Tag-804 yielded significantly lower tumor signal intensity, although its imaging contrast remained comparable due to proportionally reduced background fluorescence. Despite differences in dye net charge, no substantial variation in kidney retention was observed over 24 h. Microscopic analysis revealed distinct renal handling: 7D12–FNIR-Tag-766 showed partial endosomal internalization within proximal tubule cells, while 7D12–FNIR-Tag-1.0 remained primarily luminal.

**Conclusions:**

FNIR-Tag–labeled Nbs enable effective tumor visualization as early as 1 h post-injection. Although overall pharmacokinetics were comparable across dyes, differences in tissue uptake, intrinsic brightness, and compatibility with imaging system optics can influence detection sensitivity and should inform dye selection for clinical imaging applications.

## Introduction

Over the past two decades, Nanobodies^™^ (Nbs), single-domain antibody fragments derived from camelid heavy-chain-only antibodies[1], have emerged as highly promising platform for *in vivo* molecular imaging.[2–7] Owing to their small size (~15 kDa), high affinity, rapid tissue penetration, and fast systemic clearance, Nbs are particularly well-suited for rapid and specific imaging with high target-to-background ratios. These properties have led to Nbs being widely investigated across a range of medical imaging modalities, including nuclear and optical techniques.[4, 5, 7]

In the context of optical imaging applications such as fluorescence-guided surgery, Nbs are conjugated to near-infrared (NIR) fluorescent dyes, allowing their use as targeted contrast agents. Several studies have demonstrated that fluorescently labeled Nbs can effectively highlight tumor lesions in various mouse models and canine patients.[8–13] However, it has also become evident that the choice of fluorescent dye can significantly influence the pharmacokinetics of Nb-based tracers. Even subtle changes in charge, hydrophobicity, or overall molecular structure of the dye can alter serum protein binding, non-specific tissue uptake, and clearance mechanisms.[14–17] Such dye-induced modifications in pharmacokinetics can ultimately compromise imaging contrast and the reliability of imaging outcomes.[17, 18]

**Figure 1:**
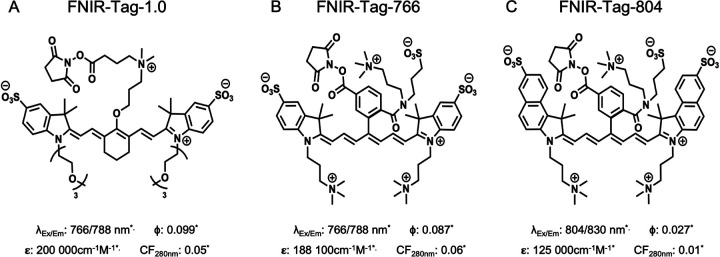
Molecular structure of FNIR-Tag-1.0 (A), FNIR-Tag-766 (B) and FNIR-Tag-804 (C) with their previously reported photophysical properties*[19–21]. λ_Ex/Em_ = Maximal emission and excitation wavelength, ɸ = Quantum yield; **ε** = Molar extinction coefficient at the maximal absorption wavelength; CF_280nm_ = Correction factor applied for absorbance at 280 nm when calculating protein concentration.

Previous studies have shown that the dye FNIR-Tag-1.0 (formerly referred to simply as FNIR-Tag) (Figure 1A) exerts minimal impact on the pharmacological behavior of Nb-tracers, in contrast to more commonly used dyes such as IRDye800CW or ZW800–1.[16, 19] FNIR-Tag-1.0 is a heptamethine dye with a stable C4′-O-alkyl group and two triethylene glycol chains at the indolenine nitrogen positions. This structure confers high brightness and reduces non-specific interactions and aggregation when conjugated to Nbs or other targeting moieties.[16, 19] More recently, pyridinum benzoxazole salts have been introduced as precursors to improve the yield and diversify the modular assembly of heptamethine dyes. This strategy led to the development of two novel dyes, FNIR-766 and FNIR-804, which are highly charged, yet overall surface-neutral, and exhibit excellent properties for *in vivo* imaging as was shown in combination with monoclonal antibodies (Figure 1B, C).[20, 21]

In the present study, we assessed and compared the impact of these three FNIR-Tag derivatives on the pharmacological behavior and tumor imaging performance of an EGFR-targeted Nb in a subcutaneous tumor model.

## Materials and Methods

### Reagents and materials

All reagents were purchased and used as obtained from Merck Sigma Aldrich (Burlington, MA, USA) or Thermo Fisher Scientific (Waltham, MA, USA), unless stated otherwise. PBS used for protein work was freshly prepared by dissolving tablets as recommended by the supplier (Millipore Sigma, Burlington, MA, USA). The synthesis of FNIR-Tag-1.0, FNIR-Tag-766 and FNIR-Tag-804 as NHS-ester is described elsewhere.[19–21] Chemical identification and purity were verified using reversed-phase high-performance liquid chromatography (RP-HPLC) analysis, nuclear magnetic resonance (NMR) analysis and electrospray high-resolution mass spectrometry (ESI/HRMS), as previously reported.[19–21]

### Production and fluorescent labeling of the anti-EGFR Nb 7D12

The anti-EGFR Nb 7D12[22], containing a carboxyterminal hexahistidine tag, was produced by the VIB Protein Core facility (Ghent, Belgium) in *Pichia pastoris* according to a standard 6 L-fermentation protocol[11, 23] and purified via immobilized metal affinity chromatography and size-exclusion chromatography. The Nb was stored at −20°C in aliquots of 1 mL in PBS at a concentration of 4.68 mg/mL until further use.

Nb 7D12 was randomly conjugated to the fluorescent dyes FNIR-Tag-1.0, FNIR-Tag-766, or FNIR-Tag-804 via NHS ester chemistry, targeting the primary amines on lysine residues of the Nb.[16] In brief, 3 mg of Nb 7D12 (900 μL) was buffered to pH 8.3–8.5 with K_2_HPO_4_ buffer (100 μL, 1 M) and a 4-fold molar excess of the dye of interest, dissolved at 20 mg/mL in DMSO, was added. The resulting mixture was incubated for 2 h at room temperature, while protected from light exposure. After incubation, the reaction was quenched by addition of HCl (20 μL, 1 M). The reaction mixture was immediately purified by size-exclusion chromatography (SEC) on a NGC chromatography system (Bio-Rad Laboratories, Hercules, CA, USA) equipped with a Superdex 75 Increase 10/300 GL column (GE Healthcare, Chicago, IL, USA) and using PBS as elution buffer (0.8 mL/min). Detection was performed at 280 nm for the Nb and at 766 or 800 nm for the respective dyes. Relevant fractions were collected in light-protecting vials. Quality control (QC) was performed by analytical SEC (50 μg of Nb in 250 μL PBS) using a Superdex 75 10/300 GL column (GE Healthcare) with PBS as elution buffer at 0.6 mL/min. The concentration of the fluorescent Nb conjugates and the degree of labeling (DOL) were determined by spectrophotometry (NanoDrop 2000, Thermo Scientific, Wilmington, DE, USA), applying a correction for absorption of the dyes at 280 nm as indicated in Figure 1. The spectral characteristics of the fluorescent Nb conjugates, dissolved in PBS at a dye concentration of 10 μM, were recorded in a quartz cuvette using a spectrofluorophotometer (RF-6000, Shimadzu, Kyoto, Japan). The excitation spectrum from 200 to 900 nm in 1 nm steps was recorded at the theoretical maximal emission wavelength of each dye. The highest excitation wavelength was used to record the corresponding emission spectrum. The spectra were normalized to the highest recorded excitation or emission signal.

Finally, freshly prepared samples of each fluorescent Nb conjugate (10 μM based on fluorescence concentration) were imaged with the Fluobeam800 v2.2 (FluOptics, Grenoble, France) with an acquisition time of 10 ms to compare the fluorescent intensity of the conjugates in relation to the imaging system used for the *in vivo* experiments.

### Functionality assessment of fluorescently-labeled anti-EGFR Nbs

The binding characteristics of the Nb conjugates were determined using surface plasmon resonance (SPR, Biacore T200, Uppsala, Sweden) and compared to the values obtained for the unconjugated Nb. Human EGFR/His (10 μg/mL, Sino Biological, Beijing, China) was immobilized on a series S Sensor Chip CM5 (Cytiva, Hoegaarden, Belgium). Test samples were then injected consecutively in three-fold serial dilutions (ranging from 250 to 0.343 nM). The association step was set at 100s, and the dissociation step at 600s. Regeneration was performed during 20s at 30 μL/min using 0.1 M glycine at pH2.5 followed by a stabilization period of 180s. The equilibrium dissociation constant (K_D_) and rate kinetic constants (k_on_ and k_off_) were determined by mathematical fitting using the ‘*1:1 binding with drift and RI2*’ model proposed by the Biacore Evaluation Software (Cytiva).

### Cell culture

FaDu cells were obtained from the American Type Culture Collection (ATCC, Manassas, VA, USA) and cultured in MEM medium (Gibco Life Technologies, Waltham, MA, USA) supplemented with 10% fetal bovine serum (FBS, Pan Biotech, Aidenbach, Germay) and 1% Pen/Strep (Gibco Life Technologies) at 37 °C in a humidified atmosphere with 5% CO2. Upon reaching confluency, they were split 1:10 following dissociation with TrypLE^™^ Express Enzyme (1x, Gibco Life Technologies).

### Animal ethics and welfare

All animal experiments were performed in accordance with the European guidelines and the Belgian national law for animal experimentation under the license LA1230272 and were approved by the ethical commission for animal experimentation of the Vrije Universiteit Brussel (20-272-7 and 22-272-35). Naïve female athymic nude Foxn1nu/nu mice (5 weeks old; 20–30 g; Charles River Laboratories, L’Abresle, France) were housed in individually ventilated cages at 19–24°C in 40–60% humidity with a light/dark cycle of 14/10 h. Animals had *ad libitum* access to water and low fluorescent food pellets (Teklad 2016, Harlan Laboratories, IN, USA). Mice were checked daily to monitor their body condition score, physical appearance, and behavior. Upon subcutaneous tumor inoculation, mice were weighed every 2–3 days and the size of their tumor was measured using a digital caliper. Predetermined humane endpoints were as follows: general abnormal behavior, abnormal posture, weight loss of ≥20% as compared to the moment of tumor inoculation, tumor size ≥ 2000 mm^3^, severe ulceration of the tumor or severely impacted mobility. Mice were killed via cervical dislocation or using an intraperitoneal injection of pentobarbital (1 g/kg; Dolethal^®^ Vetoquinol, France). During imaging procedures, injections and euthanasia, mice were kept under isoflurane-induced anesthesia (5% induction, 2% maintenance; oxygen flow rate between 0.5 and 1.5 L/min, IsoFlo, Abbott, Chicago, IL, USA).

### *In vivo* and *ex vivo* biodistribution of fluorescently-labeled anti-EGFR Nbs

To investigate the biodistribution and tumor targeting of the conjugates, 1.5×10^6^ FaDu cells were inoculated above the right hind limb and tumors were allowed to grow until a size of approximately 200 mm^3^. The mice were randomly allocated into three groups (n=5) each and were administered via intravenous tail vein injection 2 nmol (200 μL, based on fluorescent dye concentration) of 7D12-FNIR-Tag-1.0, 7D12-FNIR-Tag-766, or 7D12-FNIR-Tag-804. Fluorescent imaging of the dorsal and ventral side of the mice was performed 1 h post-injection in a dark setting using the Fluobeam800 (FluOptics) with acquisition times between 25 and 300 ms. Corresponding white light images were recorded at 1000 ms under LED illumination. After imaging, tumor, muscle, liver, lungs, intestines, kidneys, heart, spleen, and pancreas were collected for *ex vivo* fluorescence imaging. Fluorescent images were superimposed on the corresponding white light images for anatomical localization and the fluorescent intensity within regions of interest (ROIs) was quantified using ImageJ software (version 2.9.0/1.53t, NIH, Bethesda, MD, USA). On the *in vivo* images, ROIs were drawn at the level of the tumor, contralateral muscle and kidneys; on the *ex vivo* images, ROIs were drawn around each organ. The *in vivo* tumor-to-muscle ratios (TMRs) and *ex vivo* tumor-to-organ ratios were calculated by dividing the fluorescence signal of the tumor by that of the contralateral muscle or other *ex vivo* organs. The *in vivo* and *ex vivo* contrast-to-noise ratios (CNRs) were determined by subtracting the contralateral muscle or *ex vivo* organ signals from the *in vivo/ex vivo* tumor signal and dividing the result by the standard deviation of the background signal (contralateral muscle or *ex vivo* organ, respectively).

### Renal localization and retention of fluorescently-labeled Nbs

Non-tumor bearing mice (n=3 per group), injected 1 h before with 7D12-FNIR-Tag-1.0, 7D12-FNIR-Tag-766, or 7D12-FNIR-Tag-804, were killed by an intraperitoneal injection of pentobarbital and immediately transcardially perfused with 20–30 mL of ice-chilled PBS supplemented with heparin sodium salt (10 IU/ml, Sigma-Aldrich, France), followed by 20 mL of 4% paraformaldehyde (PFA, Life Technologies). Kidneys were excised and immersed in 4% PFA at 4°C. After overnight incubation, the kidneys were dehydrated and embedded in paraffin, and coronal sections (6 μm) were prepared. Sections were first scanned with an Odyssey Infrared Imaging system (Li-COR, Lincoln, NE, USA) for mesoscopic analysis (resolution: 21 μm, intensity 6.0). Subsequently, adjacent kidney sections were stained with Hoechst 33342 (1:200 dilution, 1 mg/mL, Merck) for nuclear visualization. Fluorescence microscopy was performed using a Stellaris 8 confocal microscope (Leica Microsystems, Basel, Switzerland) at 200× magnification. Emission wavelengths of 766 and 790 nm were used for visualization of FNIR-Tag-1.0 and FNIR-Tag-766 signals, respectively (FNIR-Tag-804 signals could not be detected with the used system).

To assess the renal retention of the three fluorescent Nb-conjugates over time, additional non-tumor-bearing mice (n=3 per group) were intravenously injected with 2 nmol (200 μL) of 7D12-FNIR-Tag-1.0, 7D12-FNIR-Tag-766, or 7D12-FNIR-Tag-804 and subjected to fluorescence imaging at 1, 6 and 24 h post-injection following the same imaging protocol as described above. After the last timepoint, mice were killed by cervical dislocation, and the kidneys were collected for *ex vivo* imaging.

### Statistical analysis

All data are presented as mean ± standard deviation. Data were analyzed using GraphPad Prism (version 10.5.0). Statistical differences were assessed using one-way or two-way ANOVA tests with corrections for multiple comparisons (Dunnett’s or Šidák, respectively). P-values below 0.05 were considered significant (*p<0.05, **p<0.01, ***p<0.005, ****p<0.001).

## Results

### Characterization of the fluorescent Nb conjugates

**Figure 2:**
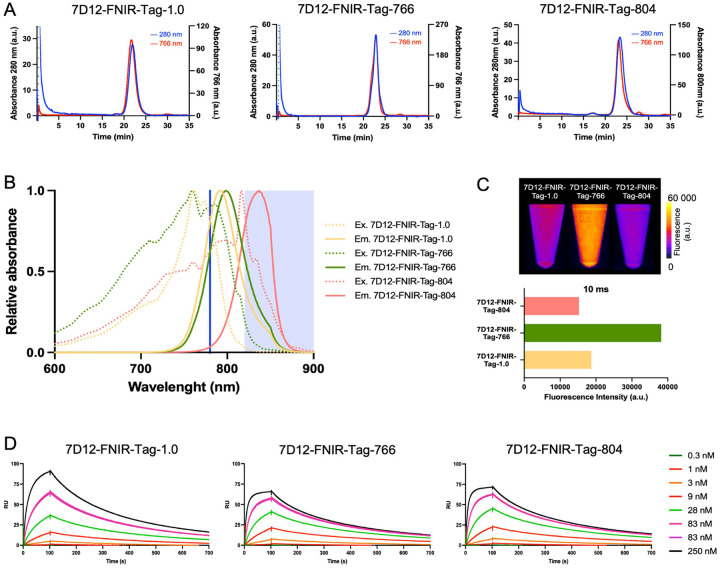
(A) Quality control of the fluorescent Nb conjugates by SEC, with absorbance at 280 nm for the Nb shown in blue and at 766/804 nm for the dye shown in red. (B) Excitation (solid lines) and emission spectra (dashed lines) of 7D12-FNIR-Tag-1.0 (orange), 7D12-FNIR-Tag-766 (green), and 7D12-FNIR-Tag-804 (red). The 780 nm laser excitation wavelength and emission filters transmitting wavelengths >820 nm employed by the FluoBeam800 imaging system are indicated in blue. (C) Fluorescence image of freshly prepared 10 μM solutions of labeled 7D12-Nb constructs, acquired using the FluoBeam800 (exposure time 12 ms) and corresponding quantification of the fluorescence intensity. (D) Binding kinetics of labeled 7D12-Nb conjugates to human EGFR/His as determined by surface plasmon resonance.

Nb 7D12 was successfully conjugated to the fluorescent dyes FNIR-Tag-1.0, FNIR-Tag-766, and FNIR-Tag-804. QC after purification showed a single peak on SEC for all, with no detectable impurities remaining (Figure 2A). The Nb conjugates had an average DOL of approximately 1 (1.1 for 7D12-FNIR-Tag-1.0, 1.3 for 7D12-FNIR-Tag-766 and 1.1 for 7D12-FNIR-Tag-804). Spectral analysis revealed that the excitation and emission profiles of the dyes remained largely consistent after conjugation to the Nb, with only a minor bathochromic shift of approximately 10 nm (Table 1, Figure S1). The spectral profiles of each Nb conjugate, in relation to the detection capabilities of the FluoBeam imaging system, are illustrated in Figure 1B. When imaged under identical conditions with this system, 7D12-FNIR-Tag-766 exhibited approximately twice the fluorescent intensity compared to 7D12-FNIR-Tag-1.0 and 7D12-FNIR-Tag-804 (Figure 2C).

SPR measurements revealed that the binding affinity of Nb 7D12 for recombinant EGFR was retained upon conjugation with FNIR-Tag-766 or FNIR-Tag-804 (7.27 nM for 7D12-FNIR-Tag-766 and 6.93 nM for 7D12-FNIR-Tag-804 as compared to 5.36 nM for the unconjugated 7D12 Nb) (Table 1). A small decrease in affinity (20.86 nM) was, however, observed for 7D12-FNIR-Tag-1.0 in consequence of a slightly slower association binding rate constant (Figure 2D).

### *In vivo* imaging and *ex vivo* biodistribution

**Figure 3:**
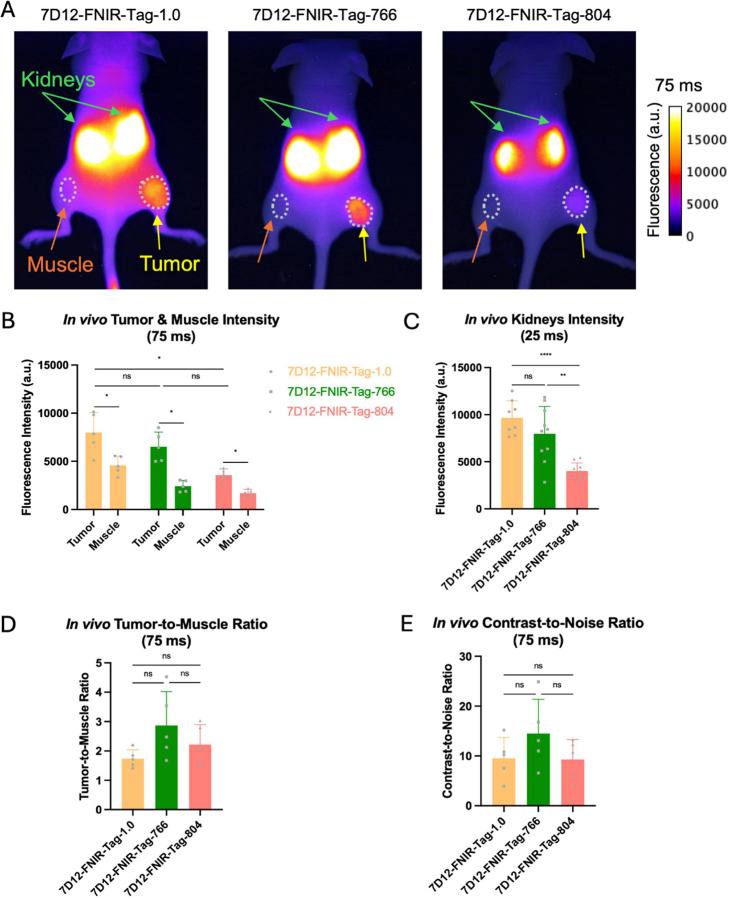
(A) Representative *in vivo* fluorescence images (exposure time 75 ms) of FaDu tumor-bearing mice (n=5), intravenously injected 1 h before with 2 nmol of 7D12-FNIR-Tag1.0, 7D12-FNIR-Tag-766 or 7D12-FNIR-Tag-804. Kidneys (green arrows), tumor (yellow arrow) and contralateral muscle (orange arrow) are indicated, as well as regions-of-interest used for quantification (gray circles). Quantification of mean fluorescent intensity at the level of the tumor and contralateral muscle (B) and the kidneys (C), as well as calculated tumor-to-muscle (D) and contrast-to-noise ratios (E). Statistical significance was defined as p < 0.05 (ns: not significant; p < 0.05; *p < 0.01; **p < 0.005; ***p < 0.0001).

**Figure 4:**
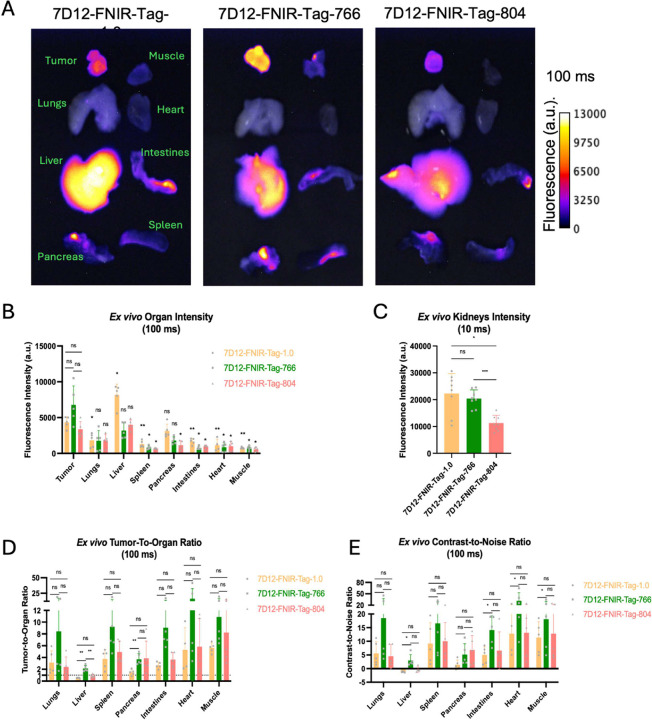
(A) Representative *ex vivo* fluorescence images (exposure time 100 ms) of major organs and tissues collected from FaDu tumor-bearing mice (n=5) intravenously injected with 2 nmol of 7D12-FNIR-Tag1.0, 7D12-FNIR-Tag-766 or 7D12-FNIR-Tag-804 1 h before. (B) Quantification of mean fluorescent intensity of the different organs. Statistical comparisons were performed both between the three tracers and between tumor and individual organs for each tracer. (D) Calculated tumor-to-organ and (E) contrast-to-noise ratios, where statistical comparisons were made between the three tracers. Statistical significance was defined as p < 0.05 (ns: not significant; p < 0.05; *p < 0.01; **p < 0.005; ***p < 0.0001).

The biodistribution and tumor-targeting of the three anti-EGFR Nb conjugates were assessed in EGFR^+^ tumor-bearing mice at 1 h post-injection (Figure 3 and 4). For direct comparison, images acquired with the same acquisition settings (i.e. exposure time) are shown and quantified.

Strong renal uptake was observed for all conjugates, reflecting rapid clearance via the kidneys (Figure 3A, green arrows). In addition, clear fluorescence was detected in the subcutaneous tumors (Figure 3A, yellow arrows). Tumor signal intensity was highest for 7D12-FNIR-Tag-1.0 and 7D12-FNIR-Tag-766, with the mean tumor fluorescence intensity significantly exceeding that of 7D12-FNIR-Tag-804. In all cases, tumor fluorescence was significantly higher than that of the contralateral muscle (Figure 3A, orange arrows, Figure 3B). 7D12-FNIR-Tag-766 provided the most favorable tumor-to-muscle and contrast-to-noise ratios, with TMR and CNR values of 2.87 ± 1.15 and 14.48 ± 6.89, respectively, compared to 1.74 ± 0.30 and 9.54 ± 4.17 for 7D12-FNIR-Tag-1.0, and 2.22 ± 0.68 and 9.28 ± 4.02 for 7D12-FNIR-Tag-804 (Figure 3D, E, no statistical differences).

*Ex vivo* analysis largely corroborated the *in vivo* findings (Figure 4). Fluorescence uptake was minimal in non-target organs, with the exception of the kidneys, which exhibited intense signals (Figure 4A, C). Compared with *in vivo* imaging, liver fluorescence became more apparent. For 7D12-FNIR-Tag-1.0 and 7D12-FNIR-Tag-804, liver signals slightly exceeded or were approximately equal to the mean tumor fluorescence, respectively, whereas for 7D12-FNIR-Tag-766, liver fluorescence remained lower than that of the tumor (Figure 4B). As a result, 7D12-FNIR-Tag-766 exhibited tumor-to-liver ratios (TLR) of 2.14 ± 0.50 and positive liver CNR values (2.97 ± 2.14), while 7D12-FNIR-Tag-1.0 and 7D12-FNIR-Tag-804 showed TLRs below 1 (7D12-FNIR-Tag-1.0: 0.54 ± 0.06, p<0.05; 7D12-FNIR-Tag-804: 0.85 ± 0.27, p<0.05) and negative liver CNRs (7D12-FNIR-Tag-1.0: −1.22 ± 0.20, p<0.05; 7D12-FNIR-Tag-804: −0.34 ± 0.98, p>0.05). In other organs, 7D12-FNIR-Tag-766 also achieved the highest tumor-to-organ ratios and CNRs, although these differences were generally not statistically significant compared to 7D12-FNIR-Tag-1.0 and 7D12-FNIR-Tag-804 (Figure 4D and E).

### Renal localization and retention of fluorescent Nb conjugates

**Figure 5:**
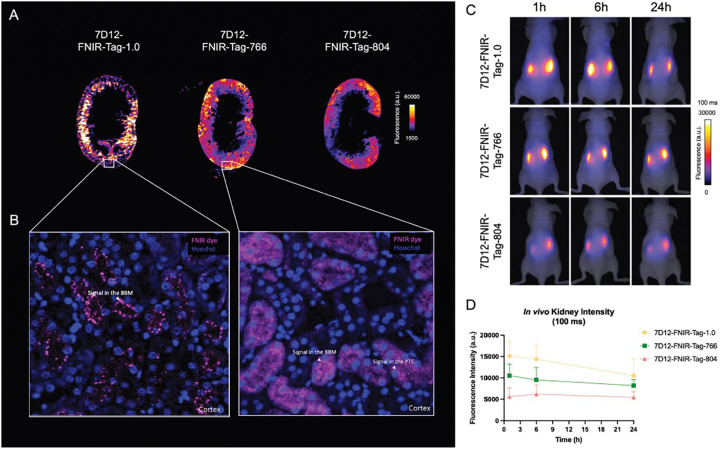
(A) Mesoscopic imaging of sagittal kidney sections from mice injected 1 h before with 7D12-Nb-FNIR-Tag-1.0, 7D12-Nb-FNIR-Tag-766 or 7D12-Nb-FNIR-Tag-804. (B) Confocal imaging of kidney sections with 7D12-Nb-FNIR-Tag-1.0 and 7D12-Nb-FNIR-Tag-766. (C) Representative fluorescence images (exposure time 100 ms) of mice at 1, 6 and 24 h post-intravenous injection of 7D12-FNR-Tag-1.0, 7D12-FNR-Tag-766 or 7D12-Nb-FNR-Tag-804 (n=3 per group). (D) Quantification of *in vivo* renal fluorescence signal over time. (E) Quantification of *ex vivo* renal fluorescence signal 24 h post injection.

Mesoscopic fluorescence imaging of paraffin-embedded sagittal kidney sections revealed primarily accumulation in the cortical region of the kidneys for all three conjugates (Figure 5A). To further explore cellular localization, confocal microscopy was performed on kidney cortex sections at 200× magnification (Figure 5B). Due to limitations in the detection capabilities of the apparatus for signals above 800 nm, *ex vivo* microscopic imaging of the kidney sections was only performed for 7D12-Nb-FNIR-Tag-1.0 and 7D12-Nb-FNIR-Tag-766, excluding 7D12-Nb-FNIR-Tag-804. For 7D12-FNIR-Tag-1.0, fluorescence was predominantly observed on the apical membrane of proximal tubule cells, corresponding to the brush border membrane, without significant intracellular localization. In contrast, 7D12-Nb-FNIR-Tag-766 exhibited a distinct distribution pattern, with fluorescence detected not only on the brush border membrane but also within the proximal tubule cells.

Finally, to evaluate the elimination of the fluorescent Nb conjugates from the kidneys over time, mice injected with 7D12-FNIR-Tag-1.0, 7D12-FNIR-Tag-766, or 7D12-FNIR-Tag-804 were imaged repeatedly over 24 h. Renal signals showed minimal decline over this period, with no notable differences observed among the three conjugates.

## Discussion

It is increasingly recognized that fluorescent dyes can significantly influence the pharmacokinetics of molecular tracers, highlighting the importance of selecting dyes with optimized properties towards *in vivo* molecular imaging applications such as fluorescence guided surgery.[14, 16–18] The FNIR-Tag dyes used in this study have previously been shown to outperform IRDye800CW-labeled tracers due to their superior stability, higher brightness, reduced propensity to aggregation, lower non-specific binding, and decreased hepatic uptake.[16, 19–21] Nevertheless, since the overall performance of a tracer is ultimately dictated by the interplay between the dye and the targeting moiety, it remains essential to validate these characteristics when introducing a different targeting agent, particularly when its size and/or physicochemical properties differs substantially. All three tested Nb-based tracers, 7D12-FNIR-Tag-1.0, −766 and −804, exhibited overall a comparable pharmacokinetic profile, characterized by rapid tumor accumulation, swift clearance from circulation and non-target tissues/organs, and predominant renal elimination. These findings corroborate with previous reports describing Nbs as molecular imaging tracers with rapid pharmacokinetics and fast, specific tumor uptake, in contrast to mAb-based tracers, which typically require several days to achieve sufficient tumor accumulation and background clearance.[4, 21, 24, 25] The improved *in vivo* performance of FNIR-Tag-1.0 is primarily due to the incorporation of short polyethylene glycol chains, which enhances its water solubility[19], while the hydrophilicity of FNIR-Tag-766 and FNIR-Tag-804 arises from their highly charged yet overall neutral structures.[20, 21] An alternative approach that has been investigated to improve *in vivo* performance of NIR dyes, and which demonstrated favorable outcomes when combined with Nbs, involves the utilization of sterically shielded dyes such as s775z.[11, 13, 16, 26]

An inherent limitation of side-by-side comparisons of tracers labeled with different fluorescent dyes lies in the bias introduced by (mis)matches between the imaging system and the spectral characteristics of each dye.[27–29] Although FNIR-Tag-1.0 and FNIR-Tag-766 share similar excitation/emission maxima, quantum yield, and extinction coefficients, *in vitro* evaluation revealed that 7D12-FNIR-Tag-766 appeared approximately twice as bright as 7D12-FNIR-Tag-1.0 when imaged with the FluoBeam. This highlights the importance of considering the full spectral profile for assessing optimal compatibility with an imaging system, and not only peak values. Moreover, FNIR-Tag-804, which was specifically designed to mimic the spectral properties of indocyanine green (ICG) for better compatibility with clinical imaging systems optimized for ICG detection, had an *in vitro* detectability of approximately 2.5-fold lower than that of 7D12-FNIR-Tag-766 and about 20% lower than 7D12-FNIR-Tag-1.0 when imaged with the FluoBeam. This is most likely due to its substantially lower quantum yield and extinction coefficient compared to the other two dyes.[21] With the increasing availability of clinical imaging platforms optimized for dyes such as S0456 and IRDye800CW, which are used in molecular tracers approved for clinical use (e.g. pafolacianine) or under clinical evaluation and which show a slight hypsochromic shift as compared to ICG, the sensitivity for tracers incorporating dyes like FNIR-Tag-766 or FNIR-Tag-1.0 is expected to improve further. [30–32]

In consequence, quantification based solely on intensity images cannot distinguish between reduced tracer uptake and diminished sensitivity of the imaging system for that tracer. Accurate assessment of biodistribution across organs would require quantifying fluorescent signals from homogenized tissues against a standard curve generated from homogenized organs spiked with known amounts of each tracer.[14, 33] However, this approach falls beyond the scope of the present study. Therefore, TBRs, which reflect tumor visibility relative to background tissue, and CNRs, which account for image contrast in the presence of noise, were deemed more appropriate metrics for the relative comparison of tracer performance.[28]

Although 7D12-FNIR-Tag-1.0 exhibited slightly higher tumor uptake and 7D12-FNIR-Tag-766 demonstrated marginally superior TBRs and CNRs, no statistically significant differences were observed between these tracers *in vivo*. In contrast, 7D12-FNIR-Tag-804 showed a significantly reduced tumor signal relative to 7D12-FNIR-Tag-1.0. Nevertheless, its TBR and CNR remained comparable to those of 7D12-FNIR-Tag-1.0 and 7D12-FNIR-Tag-766, indicating that despite diminished absolute signal intensity, overall image contrast was maintained. *Ex vivo* analysis revealed, that in addition to the tumor, the liver exhibited noticeable uptake. For 7D12-FNIR-Tag-766, tumor signals exceeded those in the liver, resulting in the highest tumor-to-liver ratio (2.14 ± 0.50) and a positive liver CNR (2.97 ± 2.14) compared to the other two tracers where liver signals slightly surpassed tumor signals. However, further studies involving orthotopic tumor models are needed to assess the ability of this tracer to reliably detect liver metastases.

Furthermore, high retention within the renal cortex is a well-documented characteristic of Nb-based tracers and was observed for all three tracers. Interestingly, 7D12-FNIR-Tag-766, despite being detected with higher sensitivity using the FluoBeam system, exhibited lower kidney signal compared to 7D12-FNIR-Tag-1.0, suggesting reduced overall renal accumulation. At the microscopic level, 7D12-FNIR-Tag-766 appeared to be partially internalized within endosomes of proximal tubule cells, whereas 7D12-FNIR-Tag-1.0 was predominantly retained in the tubular lumen. Previous work has demonstrated that differences in metabolization mechanisms of Cy5-labeled Nbs with varying dye charges can significantly influence renal retention[15]. However, in this study, no substantial difference in kidney retention was observed between these tracers over a 24 h-period, even if the net surface charge of the FNIR-Tag-1.0 (net charge −1) and FNIR-Tag-766/−804 (net-neutral) also differs. In nuclear medicine, strategies such as co-administration of agents that inhibit proximal tubule reuptake[34], incorporation of cleavable linkers[35–37], or pre-targeting approaches[38] have been employed to mitigate renal retention, as this can represent a dose-limiting toxicity factor[39]. These strategies, however, have not yet been explored for fluorescent agents. While high renal retention may complicate detection of signals near the kidneys in small animal models[8], it is less likely to impact human fluorescence imaging applications due to the presence of perinephric fat, which attenuates renal fluorescence signals.

## Conclusion

This study demonstrated that labeling of Nbs with dyes from the FNIR-Tag series, yields NIR fluorescent tracers capable of effective *in vivo* tumor visualization as early as 1 h post-administration. Although only minor differences in the overall pharmacokinetic profile were observed among the tracers, absolute tissue uptake, intrinsic spectral properties of the dyes, and their alignment with the optical characteristics of the imaging system can influence the sensitivity with which target lesions can be detected.

### Supplementary Material

**Table 1:** Chemical, spectral and affinity characteristics of the fluorescent Nb-conjugates

Supplementary Files

This is a list of supplementary files associated with this preprint. Click to download.

- Table1.docx
- COID.Chigoho.pdf
- COID.Li.pdf
- COIMSchnermann.pdf
- COIM.Stroet.pdf
- COIS.Hernot.pdf
- COIS.Pollenus.pdf
- Supplementaryinformation.pdf
- renamedd7c04.pdf
